# Supplementary material for: A neuronal ensemble encoding adaptive choice during sensory conflict in Drosophila
Source: Nat Commun. 2021 Jul 5;12:4131. doi: 10.1038/s41467-021-24423-y (PMC8257655; doi:10.1038/s41467-021-24423-y)
Supplement: Supplementary file 1 — Supplementary Information [file 41467_2021_24423_MOESM1_ESM.pdf]

A neuronal ensemble encoding adaptive choice during sensory conflict in *Drosophila*

Preeti F. Sareen[1], Li Yan McCurdy[1,2], Michael N. Nitabach[1,3,4,*]

[1]Department of Cellular and Molecular Physiology, Yale University, New Haven, CT, USA

[2]Interdepartmental Neuroscience Program, Yale University, New Haven, CT, USA

[3]Department of Genetics, Yale University, New Haven, CT, USA

[4]Department of Neuroscience, Yale University, New Haven, CT, USA

*Corresponding author: michael.nitabach@yale.edu

Supplementary Table 1

Detailed statistics and sample size for data in main figures

Figure	Datasets compared	Statistics
Fig. 1d	w^{1118} male vs. female Preference index	Mixed-effects analysis, F(9,118)=22.46, p<0.0001
		Sidak's adjusted p: (two-tailed)
	1mM male vs. female (n=10)	0.9968
	10mM male vs. female (n=10)	>0.9999
	50mM male vs. female (n=20)	0.1784
	100mM male vs. female (n=27)	0.5552
	500mM male vs. female (n=10)	0.8233
Fig. 1e	w^{1118} Preference index, Group size (n=77)	Pearson's r^2=0.05655, p=0.0373 (two-tailed)
Fig. 1f	w^{1118} Preference index, % ate (n=77)	Pearson's r^2=0.225, p<0.0001 (two-tailed)
Fig. 1g	w^{1118} % ate, Group size (n=77)	Pearson's r^2=0.0006, p=0.8313 (two-tailed)
Fig. 1h	w^{1118} Preference index, Group size, % ate	Multiple linear regression, F(3,73)=9.393, p<0.0001

		r^2=0.278
Fig. 2a	Optogenetic Screen 20XUAS-Chrimson (Chr)<br>empty>Chr (n=30)	One-way ANOVA,<br>F(40,358)=5.397,<br>p<0.0001
		Dunnett's adjusted p:<br>(two-tailed)
	empty>Chr vs. Akh>Chr (n=10)	0.9996
	**empty>Chr vs. AstA>Chr** (n=10)	**<0.0001**
	empty>Chr vs. Crz>Chr (n=10)	0.9990
	empty>Chr vs. DH44>Chr (n=10)	0.1302
	empty>Chr vs. Lk>Chr (n=10)	0.9997
	**empty>Chr vs. NPF>Chr** (n=10)	**<0.0001**
	empty>Chr vs. Proctolin>Chr (n=10)	0.9997
	empty>Chr vs. sNPF>Chr (n=10)	0.9983
	empty>Chr vs. Tk>Chr (n=10)	0.9993
	empty>Chr vs. TH>Chr (n=10)	0.9983
	empty>Chr vs. PPL1 (504B)>Chr (n=10)	0.9998
	empty>Chr vs. PPL1 (65B)>Chr (n=10)	0.9996
	empty>Chr vs. PAM (58E02)>Chr (n=10)	0.9993
	empty>Chr vs. OA/TA Tdc>Chr (n=10)	>0.9999
	empty>Chr vs. Ser/Trh>Chr (n=10)	0.9997
	**empty>Chr vs. γ2α'1>Chr** (n=10)	**<0.0001**

	empty>Chr vs. α3>Chr (n=10)	0.8644
	empty>Chr vs. γ1-pedc>Chr (n=10)	0.9986
	empty>Chr vs. α’2α2>Chr (n=10)	0.9997
	empty>Chr vs. α’2α2,γ2α’1>Chr (n=10)	0.9995
	**empty>Chr vs. α1>Chr** (n=10)	**0.0070**
	empty>Chr vs. β1>Chr (n=10)	0.1241
	empty>Chr vs. β1β2>Chr (n=10)	0.9924
	empty>Chr vs. γ5>Chr (n=10)	0.9997
	empty>Chr vs. β’2a>Chr (n=6)	0.9983
	empty>Chr vs. γ4,γ4<γ1γ2>Chr (n=10)	0.9982
	empty>Chr vs. γ3>Chr (n=10)	0.9990
	empty>Chr vs. allKC 10B>Chr (n=10)	0.9988
	empty>Chr vs. α/β 8B>Chr (n=8)	0.9988
	empty>Chr vs. α/β c739>Chr (n=10)	0.9988
	empty>Chr vs. α'/β' 5B>Chr (n=10)	0.9997
	empty>Chr vs. γ-m 131B>Chr (n=8)	0.3762
	empty>Chr vs. FBl4,6 ss20>Chr (n=15)	0.9997
	empty>Chr vs. FBl3,4,6 ss208>Chr (n=10)	0.9990
	empty>Chr vs. FBl3,4,6 ss225>Chr (n=10)	0.9993
	empty>Chr vs. FBl6 c205>Chr (n=10)	0.9777
	empty>Chr vs. FBl2,8,9 R89E07>Chr (n=10)	0.6555
	empty>Chr vs. FBl5,8,9 R38E07>Chr (n=10)	0.9994

	empty>Chr vs. ventral FB R58F03>Chr (n=10)<br>empty>Chr vs. FBl1,2 R52G12>Chr (n=10)	0.3548<br>0.9987
Fig. 2a	Optogenetic Screen 20XUAS-GtACR1 (Gt)<br>empty>Gt (n=30)	One-way ANOVA, $F(10,129)=7.719$, $p<0.0001$
		Dunnett's adjusted p: (two-tailed)
	**empty>Gt vs. AstA>Gt** (n=10)<br>**empty>Gt vs. DH44>Gt** (n=10)<br>**empty>Gt vs. Lk>Gt** (n=10)<br>empty>Gt vs. NPF>Gt (n=10)<br>empty>Gt vs. γ2α'1>Gt (n=10)<br>**empty>Gt vs. α3>Gt1** (n=10)<br>empty>Gt vs. α1>Gt (n=10)<br>empty>Gt vs. β1>Gt (n=10)<br>**empty>Gt vs. FBl6 c205>Gt** (n=10)<br>empty>Gt vs. FBl2,8,9 89E07>Gt (n=20)	**0.0004**<br>**0.0023**<br>**0.0081**<br>0.4307<br>>0.9999<br>**<0.0001**<br>0.9628<br>0.9996<br>**0.0002**<br>0.2042
Fig. 2b	Lk (left panel)	One-way ANOVA, $F(4,84)=8.136$, $p<0.0001$
		Sidak's adjusted p: (two-tailed)

	empty>Chr (n=10) (n=10) vs. Lk>Chr (n=20)<br>empty>Chr (n=10) vs. Lk>UAS-Chr;UAS-DH44^{RNAi} (n=30)<br>**empty>Gt** (n=10) **vs. Lk>Gt** (n=19)	0.2664<br>0.3550<br>**0.0005**
Fig. 2b	Lk (right panel)<br>RNAi ctrl = Lk-GAL4>UAS-Valium (n=20)	One-way ANOVA, F(7,106)=1.973, p=0.0655
	RNAi ctrl vs. AstA-R1^{RNAi} (n=14)<br>RNAi ctrl vs. DH44-R1^{RNAi} (n=10)<br>RNAi ctrl vs. NPFR^{RNAi} (n=10)<br>RNAi ctrl vs. Dop1R1^{RNAi} (n=20)<br>RNAi ctrl vs. Dop1R2^{RNAi} (n=10)<br>RNAi ctrl vs. Dop2R^{RNAi} (n=20)<br>RNAi ctrl vs. DopEcR^{RNAi} (n=10)	Multiple comparisons not carried out since ANOVA is not significant
Fig. 2c	AstA (left panel)	One-way ANOVA, F(4,75)=61.57, p<0.0001
		Sidak's adjusted p: (two-tailed)
	**empty>Chr** (n=10) **vs. Chr** (n=20)<br>empty>Chr (n=10) vs. AstA>UAS-Chr;UAS-AstA^{RNAi} (n=20)<br>**empty>Gt** (n=10) **vs. AstA>Gt** (n=20)	**<0.0001**<br>0.9814<br>**<0.0001**

Fig. 2c	AstA (right panel)<br>RNAi ctrl = AstA-GAL4>UAS-Valium (n=20)	One-way ANOVA,<br>F(7,90)=4.368,<br>p=0.0003
		Dunnett's adjusted p:<br>(two-tailed)
	RNAi ctrl vs. DH44-R1^{RNAi} (n=10)	0.9530
	RNAi ctrl vs. Lkr^{RNAi} (n=5)	0.2010
	RNAi ctrl vs. NPFR^{RNAi} (n=13)	0.5986
	**RNAi ctrl vs. Dop1R1^{RNAi}** (n=20)	**0.0005**
	RNAi ctrl vs. Dop1R2^{RNAi} (n=10)	0.9998
	RNAi ctrl vs. Dop2R^{RNAi} (n=10)	0.9979
	RNAi ctrl vs. DopEcR^{RNAi} (n=10)	0.9975
Fig. 2d	NPF (left panel)	One-way ANOVA,<br>F(5,89)=11.81,<br>p<0.0001
		Sidak's adjusted p:<br>(two-tailed)
	**empty>Chr** (n=10) **vs. NPF>Chr** (n=20)	**0.0002**
	empty>Chr (n=10) vs. NPF>UAS-Chr;UAS-NPF^{RNAi} (n=25)	0.9855
	empty>Gt (n=10) vs. NPF>Gt (n=20)	0.1928

Fig. 2d	NPF (right panel)<br><br>RNAi ctrl = NPF-GAL4>UAS-Valium (n=20)	One-way ANOVA,<br>F(7,127)=3.657,<br>p=0.0012
		Dunnett's adjusted p:<br>(two-tailed)
	RNAi ctrl vs. AstA-R1^{RNAi} (n=15)<br>RNAi ctrl vs. DH44-R1^{RNAi} (n=10)<br>**RNAi ctrl vs. Lkr ^{RNAi}** (n=20)<br>**RNAi ctrl vs. Dop1R1^{RNAi}** (n=20)<br>RNAi ctrl vs. Dop1R2^{RNAi} (n=20)<br>RNAi ctrl vs. Dop2R^{RNAi} (n=20)<br>RNAi ctrl vs. DopEcR^{RNAi} (n=10)	0.4148<br>0.9972<br>**0.0188**<br>**0.0026**<br>0.1588<br>0.9212<br>0.9910
Fig. 2e	DH44 (left panel)	One-way ANOVA,<br>F(4,75)=10.54,<br>p<0.0001
		Sidak's adjusted p:<br>(two-tailed)
	empty>Chr (n=10) vs. DH44>Chr (n=20)<br>empty>Chr (n=10) vs. DH44>UAS-Chr;UAS-DH44^{RNAi} (n=20)<br>**empty>Gt** (n=10) **vs. DH44>Gt** (n=20)	0.1591<br>0.9807<br>**<0.0001**

Fig. 2e	DH44 (right panel)<br>RNAi ctrl = DH44-GAL4>UAS-Valium (n=20)	One-way ANOVA,<br>$F(7,141)=5.56$,<br>$p<0.0001$
		Dunnett's adjusted p:<br>(two-tailed)
	RNAi ctrl vs. DH44>AstA-R1^{RNAi} (n=20)<br>RNAi ctrl vs. DH44>Lkr^{RNAi} (n=20)<br>RNAi ctrl vs. DH44>NPFR^{RNAi} (n=19)<br>RNAi ctrl vs. DH44>Dop1R1^{RNAi} (n=20)<br>RNAi ctrl vs. DH44>Dop1R2^{RNAi} (n=10)<br>RNAi ctrl vs. DH44>Dop2R^{RNAi} (n=20)<br>**RNAi ctrl vs. DH44>DopEcR^{RNAi}** (n=20)	0.7806<br>0.6273<br>0.9997<br>0.9998<br>0.9996<br>0.9952<br>**0.0001**
Fig. 3a	c205 (left panel)	One-way ANOVA,<br>$F(14,318)=3.315$,<br>$p<0.0001$<br>Sidak's adjusted p:<br>(two-tailed)
	empty>Chr (n=26) vs. c205>Chr (n=45)<br>**empty>Gt** (n=20) **vs. c205>Gt** (n=20)<br>empty>Gt (n=20) vs. 84C10>GAL80+c205>Gt (n=10)<br>**c205>Gt** (n=20) **vs. 84C10>GAL80+c205>Gt** (n=10)	0.2150<br>**<0.0001**<br>0.7195<br>**0.0008**
	c205 (right panel)<br>RNAi ctrl = c205-GAL4>UAS-Valium (n=47)	Kruskal-Wallis<br>stat=40.85, $p<0.0001$

		Dunn's adjusted p: (two-tailed)
	RNAi ctrl vs. $AstA^{RNAi}$ (n=20)	0.2550
	**RNAi ctrl vs. $AstA\text{-}R1^{RNAi}$** (n=20)	**0.0131**
	RNAi ctrl vs. $DH44^{RNAi}$ (n=20)	0.1245
	**RNAi ctrl vs. $DH44\text{-}R1^{RNAi}$** (n=20)	**0.0001**
	RNAi ctrl vs. Lk^{RNAi} (n=20)	0.9999
	**RNAi ctrl vs. Lkr^{RNAi}** (n=20)	**0.0011**
	RNAi ctrl vs. NPF^{RNAi} (n=20)	0.9999
	RNAi ctrl vs. $NPFR^{RNAi}$ (n=40)	0.9999
	RNAi ctrl vs. $Dop1R1^{RNAi}$ (n=20)	0.9999
	RNAi ctrl vs. $Dop1R2^{RNAi}$ (n=20)	0.9999
	RNAi ctrl vs. $Dop2R^{RNAi}$ (n=20)	0.6954
	RNAi ctrl vs. $DopEcR^{RNAi}$ (n=31)	0.9999
Fig. 3b	c205 % ate	Kruskal-Wallis stat=49.98, p<0.0001
		Dunn's adjusted p: (two-tailed)
	**c205>Chr deprived** (n=29) **vs. fed** (n=10)	**<0.0001**
	**c205>Gt deprived** (n=20) **vs. fed** (n=10)	**<0.0001**
Fig. 3c	Food intake<br>empty>Chr (n=10) c205>Chr (n=8)	Kruskal-Wallis stat=3.022, p=0.6966

Fig. 3d	Food intake<br>empty>Gt (n=7), c205>Gt (n=7)	Kruskal-Wallis<br>stat=4.189, p=0.5225
Fig. 3e	Place PI<br>empty>Chr (n=8), c205>Chr (n=20)<br>empty>Gt (n=7), c205>Gt (n=8)	One-way ANOVA,<br>F(3,39)=2.284, p=0.094
Fig. 4d	84C10 Peak $\Delta R/R_0$	Kruskal-Wallis<br>stat=37.79, p<0.0001
		Two-tailed Wilcoxon matched-pairs or Paired t test p:
	**naïveDeprived sweet vs. naïveDeprived bittersweet** (n=10)	**0.0488**
	naïveFed sweet vs. naïveFed bittersweet (n=12)	0.9821 (t=0.02289,df=11)
	**choseSweet sweet vs. choseSweet bittersweet** (n=8)	**0.0078**
	**choseBittersweet sweet vs. choseBittersweet bittersweet** (n=9)	**0.0043** (t=3.937,df=8)
	choseNeither sweet vs. choseNeither bittersweet (n=10)	0.7755 (t=0.2939, df=9)
Fig. 5a	Preference index	One-way ANOVA<br>F(3,30)=9.21, p=0.0002
		Sidak's adjusted p:<br>(two-tailed)
	**c205>w1118 deprived** (n=6) **vs. c205>Gt** (n=12)	**0.0095**
	**84C10>w1118 deprived** (n=6) **vs. 84C10>Gt** (n=10)	**0.0014**

Supplementary Table 2

Source for all fly genotypes used

Figure	Genotype	Source
Fig. 2, 3	empty = Empty split-GAL4	FlyLight Robot ID: 3019156
Fig. 2, 3	Chr = 20XUAS-CsChrimson (X)	RRID:BDSC_55134
Fig. 2, 3	20XUAS-CsChrimson (II) for Chr;RNAi experiments	RRID:BDSC_55136
Fig. 2, 3	Gt = 20XUAS-GtACR1 (III)	Rebecca Yang (Duke), A. Claridge-Chang (Duke-NUS)
Fig. 2	Akh-GAL4	RRID:BDSC_25684
Fig. 2	AstA-GAL4	RRID:BDSC_51979
Fig. 2	Crz-GAL4	RRID:BDSC_51976
Fig. 2	DH44-GAL4	RRID:BDSC_51987
Fig. 2	Lk-GAL4	RRID:BDSC_51993
Fig. 2	NPF-GAL4	RRID:BDSC_25682
Fig. 2	Proctolin-GAL4	RRID:BDSC_51972
Fig. 2	sNPF-GAL4	RRID:BDSC_51991
Fig. 2	Tk-GAL4	RRID:BDSC_51973
Fig. 2	TH-GAL4 (ple-GAL4)	RRID:BDSC_8848
Fig. 2	(PPL1) MB504B-GAL4	RRID:BDSC_68329
Fig. 2	(PPL1) MB065B-GAL4	RRID:BDSC_68281
Fig. 2	(PAM) 58E02-GAL4	RRID:BDSC_41347
Fig. 2	Tdc-GAL4	RRID:BDSC_9313

Fig. 2	Trh-GAL4	RRID:BDSC_38388
Fig. 2	(PPL1-γ2α’1) MB296B-GAL4 [1, 2]	RRID:BDSC_68308
Fig. 2	(PPL1-α3) MB630B-GAL4 [2]	RRID:BDSC_68334
Fig. 2	(PPL1-γ1-pedc) MB320C-GAL4 [2]	RRID:BDSC_68253
Fig. 2	(PPL1-α’2α2) MB058B-GAL4 [1]	RRID:BDSC_68278
Fig. 2	(PPL1-α’2α2, γ2α’1) MB099C-GAL4 [2]	RRID:BDSC_68290
Fig. 2	(PAM-α1) MB043C-GAL4 [1, 2]	RRID:BDSC_68363
Fig. 2	(PAM-β1) MB063B-GAL4 [1, 2]	RRID:BDSC_68248
Fig. 2	(PAM-β1β2) MB213B-GAL4 [1, 2]	RRID:BDSC_68273
Fig. 2	(PAM-γ5) MB315C-GAL4 [1, 2]	RRID:BDSC_68316
Fig. 2	(PAM-β’2a) MB109B-GAL4 [1, 2]	RRID:BDSC_68261
Fig. 2	(PAM-γ4,γ4< γ1γ2) MB312C-GAL4 [1]	RRID:BDSC_68252
Fig. 2	(PAM-γ3) MB441B-GAL4 [1]	RRID:BDSC_68251
Fig. 2	(all KC) MB010B-GAL4 [1]	FlyLight Robot ID: 2135061
Fig. 2	(α/β KC) MB008B-GAL4 [1]	FlyLight Robot ID: 2135059
Fig. 2	(α/β KC) c739-GAL4	RRID:BDSC_7362
Fig. 2	(α’/β’ KC) MB005B-GAL4 [1]	FlyLight Robot ID: 2135056
Fig. 2	(γ-m KC) MB131B-GAL4 [1]	FlyLight Robot ID: 2135179
Fig. 2	(FBl4,6) ss20-GAL4 (III)	L. Shao, U. Heberlein, FlyLight
Fig. 2	(FBl4,6) ss208-GAL4 (III)	T. Wolff, A. Jenett, G. Rubin, FlyLight
Fig. 2	(FBl4,6) ss225-GAL4 (III)	T. Wolff, A. Jenett, G. Rubin, FlyLight
Fig. 2, 3	(FBl6) c205-GAL4	RRID:BDSC_30826
Fig. 2	(FBl2,8,9) 89E07-GAL4 [3]	RRID:BDSC_40553

Fig. 2	(FBl5,8,9) 38E07-GAL4 [3]	RRID:BDSC_50007
Fig. 2	(ventral FB) 58F03-GAL4	RRID:BDSC_39187
Fig. 2	(FBl1,2) 52G12-GAL4	RRID:BDSC_49581
Fig. 2, 3	UAS-Valium	RRID:BDSC_35786
Fig. 2, 3	UAS-Lk-RNAi [4, 5, 6, 7]	RRID:BDSC_25798
Fig. 2, 3	UAS-Lkr-RNAi [4, 6, 8]	RRID:BDSC_25936
Fig. 2, 3	UAS-AstA-RNAi [7, 9]	RRID:BDSC_25866
Fig. 2, 3	UAS-AstA-R1-RNAi [10]	RRID:BDSC_27280
Fig. 2, 3	UAS-NPF-RNAi [7, 11, 12]	RRID:BDSC_27237
Fig. 2, 3	UAS-NPFR-RNAi [6, 12, 13, 14]	RRID:BDSC_25939
Fig. 2, 3	UAS-DH44-RNAi [7, 15, 16]	RRID:BDSC_25804
Fig. 2, 3	UAS-DH44-R1-RNAi [15]	RRID:BDSC_28780
Fig. 2, 3	UAS-Dop1R1-RNAi [14]	RRID:BDSC_62193
Fig. 2, 3	UAS-Dop1R2-RNAi [14]	RRID:BDSC_65997
Fig. 2, 3	UAS-Dop2R-RNAi [14]	RRID:BDSC_26001
Fig. 2, 3	UAS-DopEcR-RNAi [14]	RRID:BDSC_31981
Fig. 3	(FBl6) 84C10-LexA	RRID:BDSC_54339
Fig. 3	8XLexAop-GAL80	RRID:BDSC_32213
Fig. 4	(FBl6) 84C10-GAL4	RRID:BDSC_48378
Fig. 4	UAS-GCaMP6f;UAS-tdTomato	D. Clark, Yale University

Supplementary Table 3

Detailed statistics and sample size for data in supplementary figures

Figure	Datasets compared	Statistics
Suppl. Fig. 1d	***w1118* 100 mM sucrose**,<br>2h (n=20), 6 h (n=10), 21 h (n=20)<br>Slope -0.1652<br>SE of slope -0.06535<br>95% CI of slope -0.03372 to -0.2966	One-way ANOVA,<br>F(2,47)=3.53, p=0.0372<br>Test for linear trend:<br>F(1, 47)=6.39, **p=0.0149**
Suppl. Fig. 1f	**CS 50 mM sucrose**<br>**5 h (n=12), 21 h (n=12)**	Two-tailed Unpaired t test<br>t=2.611, df=22, **p=0.016**
Suppl. Fig. 1j	*w1118* transitions per fly<br>first half vs. second half (n=201)	Two-tailed Paired t test<br>t=-0.84, df=200, p=0.4004
Suppl. Fig. 2b	Lk % ate<br>**Lk>Chr** (n=10) vs. **Lk>Chr;Lk-RNAi** (n=20)	Two-tailed Mann-Whitney t test<br>**p<0.0001**
Suppl. Fig. 3b	84C10-GAL4 left panel	One-way ANOVA,<br>F(3,50)=7.82, **p=0.0002**
		Sidak's adjusted p:
	84C10>Valium (n=17) vs. 84C10>Chr (n=17)<br>**84C10>Valium (n=10) vs. 84C10>Gt (n=10)**	0.1463<br>**0.0034**
	84C10-GAL4 right panel	One-way ANOVA,<br>F(3,28)=5.02, **p=0.0066**
		Dunnett's adjusted p:
	**RNAi ctrl** (n=8) **vs. AstA-R1**$^{\mathbf{RNAi}}$ (n=10)	**0.0069**

	**RNAi ctrl** (n=8) **vs. DH44-R1**^{RNAi} (n=9)<br>**RNAi ctrl** (n=8) **vs. Lkr**^{RNAi} (n=5)	**0.0417**<br>**0.0078**
Suppl. Fig. 3c	84C10-GAL4 % ate	One-way ANOVA, F(3,55)=186.1, **p<0.0001**
		Sidak's adjusted p:
	**deprived 84C10>Chr (n=17) vs. fed 84C10>Chr (n=15)**<br>**deprived 84C10>Gt (n=20) vs. fed 84C10>Gt (n=7)**	**<0.0001**<br>**<0.0001**
Suppl. Fig. 3d	84C10-GAL4 Place PI<br>84C10>*w1118* (n=8), 84C10>Chr (n=12)<br>84C10>*w1118* (n=7), 84C10>Gt (n=12)	One-way ANOVA, F(3,35)=1.95, p=0.1389
Suppl. Fig. 4b	84C10 Peak $\Delta R/R_0$ prior sweet experience<br>sweet vs. bittersweet (n=5)	Two-tailed Paired t test<br>t=0.5381, df=4, p=0.6191
Suppl. Fig. 4d	84C10 Peak $\Delta R/R_0$ prior bittersweet experience<br>sweet vs. bittersweet (n=7)	Two-tailed Paired t test :<br>t=1.258, df=6, p=0.2552
Suppl. Fig. 5a	Preference index	One-way ANOVA,<br>F(2,22)=7.41, p=0.0035<br>Dunnett's adjusted p:
	empty>Chr (n=5) vs. c205>Chr (n=10)<br>empty>Chr (n=5) vs. 84C10>Chr (n=10)	0.0634<br>0.4417

Suppl. Fig. 5b	Preference index left panel	One-way ANOVA, F(3,8)=4.37, p=0.0424 Sidak's adjusted p:
	c205>w1118 (n=3) vs. c205>Chr (n=3)	0.1867
	84C10>w1118 (n=3) vs. 84C10>Chr (n=3)	0.0873
	Preference index left panel (n=3)	One-way ANOVA, F(3,8)=0.81, p=0.52

Supplementary Figure 1

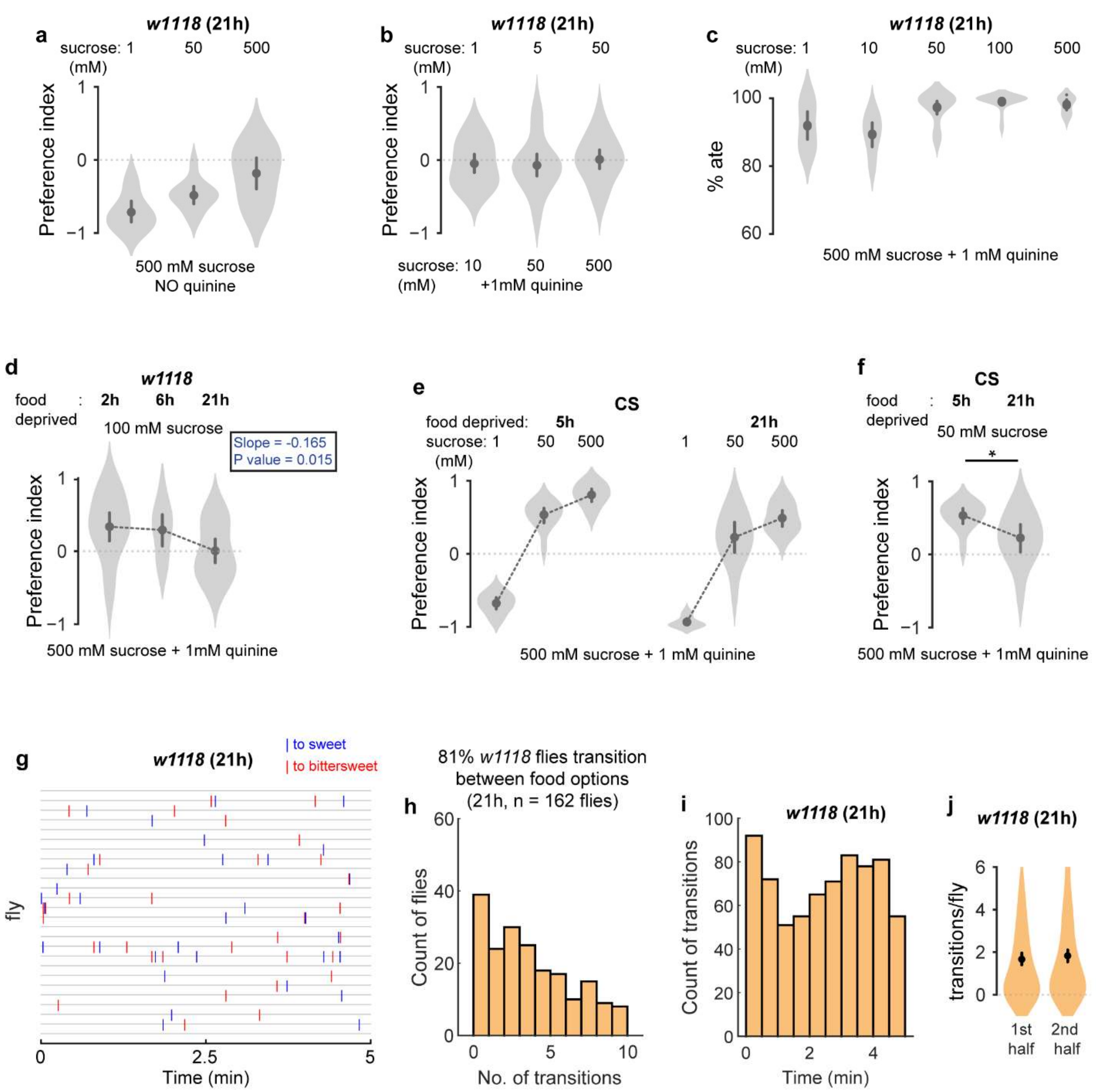

Supplementary Figure 1. Food content and hunger affect food preference. a *w1118* flies always preferred higher sucrose concentration when no quinine was present (n = 10 each). **b** Food preference depended on sucrose concentration ratio between the two food options when quinine concentration was kept constant (n = 24 each). **c** Most *w1118* flies ate after 21 h food deprivation, with almost 100% eating at the equal-preference 50 mM sucrose condition (n = 10

each except 50 mM n = 20, 100 mM n = 27). **d** Food preference in *w1118* flies shifted from sweet to equal-preference for sweet and bittersweet as food deprivation duration increased from 2 – 21 h at 100 mM sucrose vs. 500 mM sucrose + 1 mM quinine. A linear downward trend from 2 h to 21 h was statistically significant. **e** CS flies also showed shift in food preference at equal preference condition (50 mM sucrose vs. 500 mM sucrose + 1 mM quinine) with varying food deprivation duration (n = 12 each). **f** At equal-preference condition, CS flies preferred sweet after 5 h food deprivation while they showed equal-preference for sweet and bittersweet after 21 h food deprivation. **g** *w1118* fly tracking during decision task at equal-preference condition (50 mM sucrose vs. 500 mM sucrose + 1 mM quinine) showed that flies transitioned from one food patch to the other and sampled both foods multiple times throughout the assay. X-axis depicts time during the assay and y-axis shows single fly transitions over time from all flies during a sample trial. Blue depicts transition from bittersweet to sweet patch, while red depicts transition from sweet to bittersweet patch. **h** Histogram of number of flies that made a certain number of transitions. 81% of flies made at least one transition, while majority of flies (69%) made at least 2 transitions during the decision task. **i** Histogram of number of transitions made over time during the decision task. Flies transitioned throughout the task with **j** no significant difference between the number of transitions per fly during the first and the second half of the task. Plots depict mean ± 95% CI; violins show data distribution. See Supplementary Table 3 for statistics and sample size. $p<0.01$=**, $p<0.05$=*. Non-significant differences are not depicted in figures.

Supplementary Figure 2

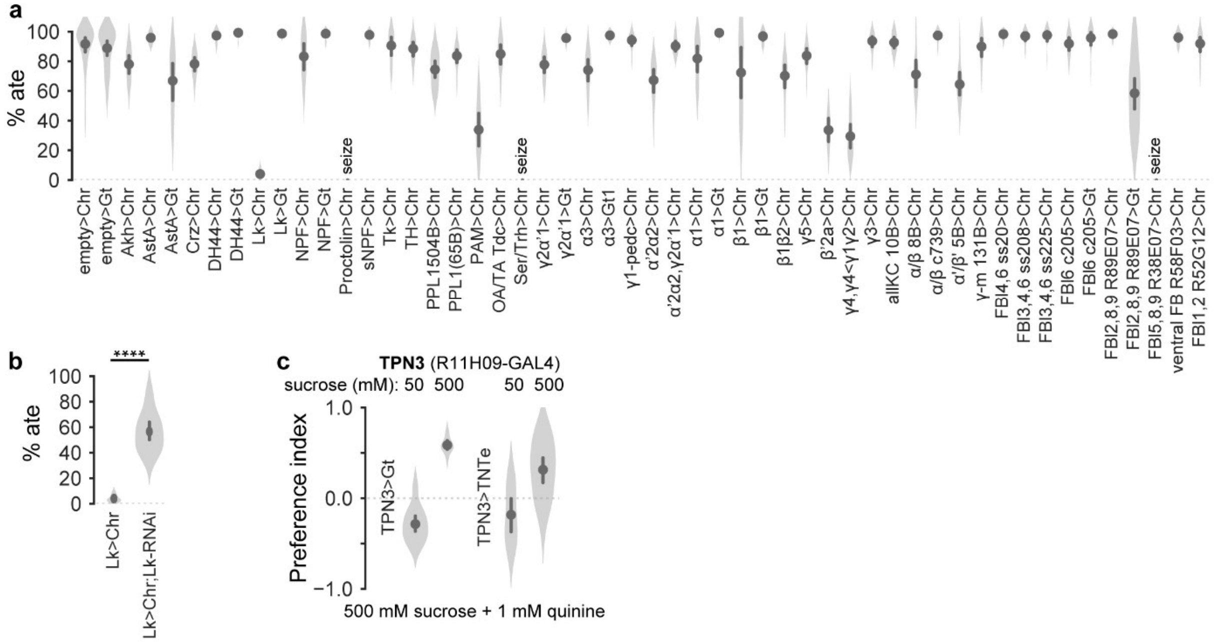

Supplementary Figure 2. Percentage of flies that ate during decision assay. a Percent of flies that ate during the optogenetic screen for all the genotypes tested. n is the same as in Figure 2a. **b** Only ~4% of the flies ate when Lk neurons were activated (Lk>Chr) and this effect was abolished (~57% ate) by knocking down Lk in the same neurons during optogenetic activation (Lk>Chr;Lk-RNAi). **c** Acute optogenetic (TPN3>Gt) and chronic inhibition (TPN3>TNTe) of second-order taste projection neurons (TPN3) did not change food preference at the equal-preference condition (50 mM, n for Gt = 20, n for TNTe = 10) or when sucrose concentration was the same in both sweet and bittersweet options (500 mM, n for Gt = 10, n for TNTe = 20). Plots depict mean ± 95% CI; violins show data distribution. See Supplementary Table 3 for statistics and sample size. p<0.0001=***.

Supplementary Figure 3

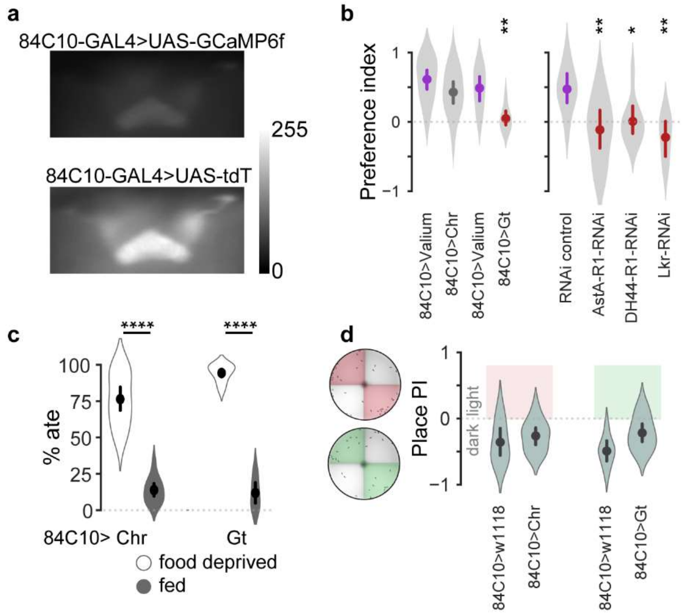

Supplementary Figure 3. 84C10-GAL4 characterization. a 84C10-GAL4 showed high baseline GCaMP6f fluorescence. Images shown are raw florescence images from the same frame without background subtraction. **b** 84C10-GAL4 had the same behavioral phenotype as c205-GAL4 **b** (left) when optogenetically activated (84C10>Chr) and inhibited (84C10>Gt) compared to appropriate controls. Flies preferred bittersweet food compared to control flies when FBl6 neurons were inhibited using 84C10-GAL4. **b** (right) Receptor RNAi knockdown of AstA, DH44 and LK in FBl6 neurons using 84C10-GAL4 had the same effect as with c205-GAL4. Flies preferred more bittersweet compared to control upon receptor RNAi knockdown. **c** Feeding was not initiated in fed flies and not inhibited in food-deprived flies on FBl6 activation or inhibition using 84C10-GAL4. **d** Neither activation nor inhibition of FBl6 was inherently rewarding or aversive since there was no significant difference in place preference without food for illuminated vs. non-illuminated sectors of the arena. Plots depict mean ± 95% CI; violins show data distribution. See Supplementary Table 3 for statistics and sample size. $p<0.00001$=****, $p<0.0001$=***, $p<0.01$=**, $p<0.05$=*.

Supplementary Figure 4

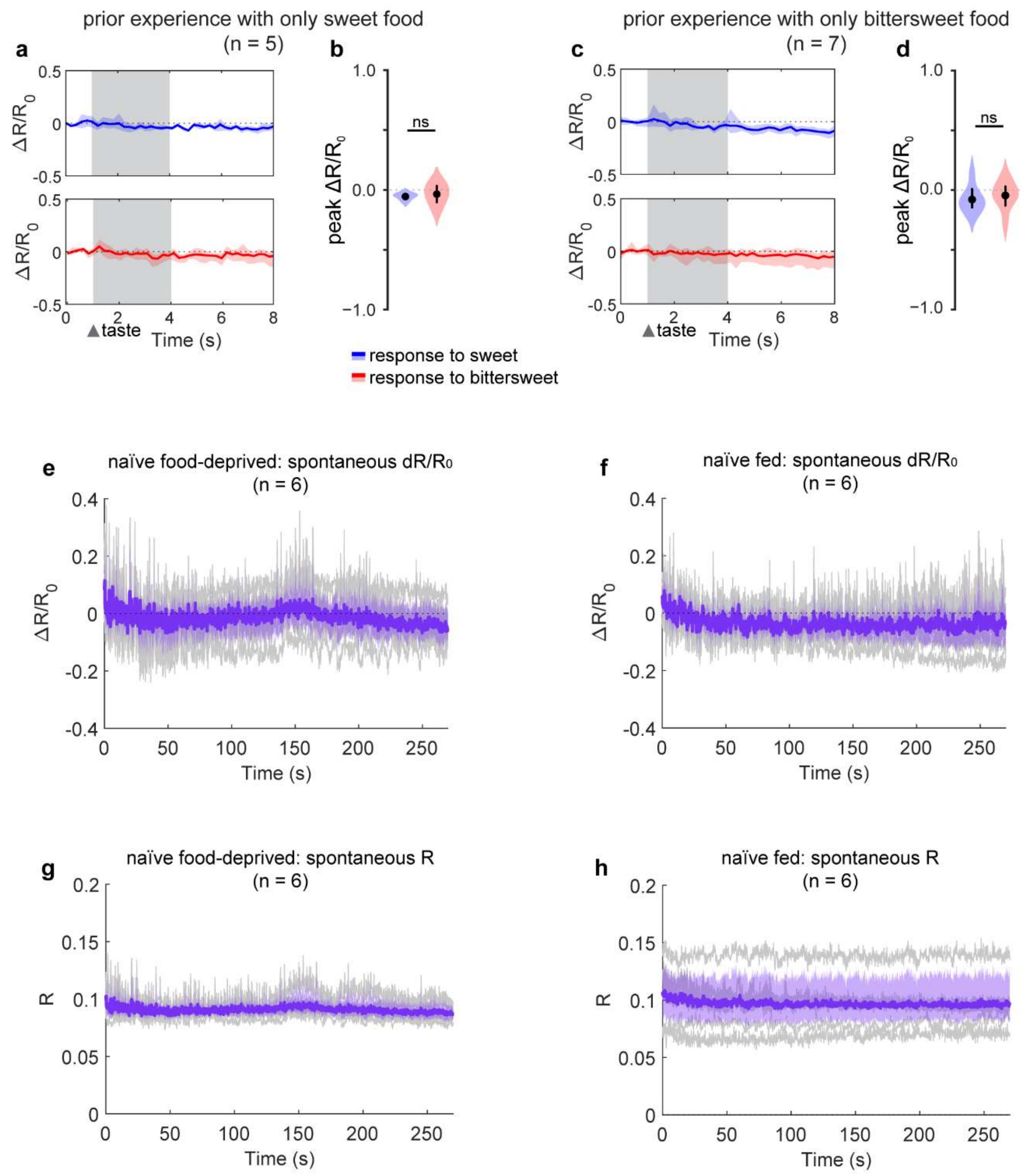

Supplementary Figure 4. FBl6 neural activity is context-dependent. a FBl6 ratiometric calcium responses to sweet and bittersweet taste stimuli, $\Delta R/R_0$, of food-deprived flies that experienced only sweet food prior to imaging. Gray background area represents taste application. Calcium activity trace depicts mean $\Delta R/R_0 \pm 95\%$ CI. **b** Peak $\Delta R/R_0$ show no significant

difference between response to experienced sweet and new bittersweet food stimulus. **c** FBl6 ratiometric calcium responses to sweet and bittersweet taste stimuli, $\Delta R/R_0$, of flies that experienced only bittersweet food prior to imaging. Gray background area represents taste application. Calcium activity trace depicts mean $\Delta R/R_0 \pm 95\%$ CI. **d** Peak $\Delta R/R_0$ show no significant difference between response to experienced bittersweet and new sweet food stimulus. **e** Spontaneous normalized FBl6 ratiometric calcium response, $\Delta R/R_0$, without any taste application in naïve food-deprived flies. **f** Spontaneous normalized FBl6 ratiometric calcium response, $\Delta R/R_0$, without any taste application in naïve fed flies. **g** Spontaneous FBl6 calcium response, R, without any taste application in naïve food-deprived flies. **h** Spontaneous FBl6 ratiometric calcium response, R, without any taste application in naïve fed flies. Points on graphs depict mean ± 95% CI, with violins depicting full data distribution. See Supplementary Table 3 for details on statistics and sample size).

Supplementary Figure 5

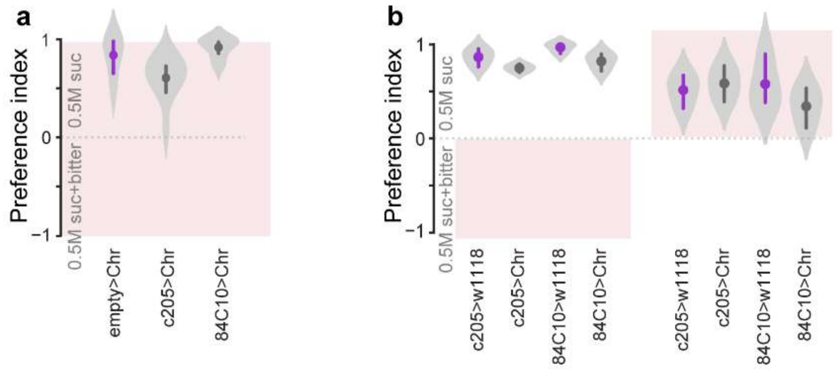

Supplementary Figure 5. Activation of FBl6 neurons in any context has no effect on food preference. a Optogenetically activating (c205>Chr and 84C10>Chr) FBl6 neurons throughout the food arena in food-deprived flies at the condition in which control flies had a strong preference for sweet food (0.5 M sucrose vs. 0.5 M sucrose + 1 m M quinine) had no effect on food preference. **b** Optogenetically activating (c205>Chr and 84C10>Chr) FBl6 neurons on only bittersweet food (left panel) in food-deprived flies at the condition in which control flies had a strong preference for sweet food (0.5 M sucrose vs. 0.5 M sucrose + 1 m M quinine) had no effect on food preference. Activating FBl6 neurons on sweet food only also did not shift preference in this condition (right panel). Plots depict mean ± 95% CI; violins show data distribution. See Supplementary Table 3 for statistics and sample size.

Supplementary References

1. Aso Y*, et al.* The neuronal architecture of the mushroom body provides a logic for associative learning. *Elife* **3**, e04577 (2014).

2. Aso Y, Rubin GM. Dopaminergic neurons write and update memories with cell-type-specific rules. *Elife* **5**, (2016).

3. Hu W*, et al.* Fan-Shaped Body Neurons in the Drosophila Brain Regulate Both Innate and Conditioned Nociceptive Avoidance. *Cell Rep* **24**, 1573-1584 (2018).

4. Cavey M, Collins B, Bertet C, Blau J. Circadian rhythms in neuronal activity propagate through output circuits. *Nat Neurosci* **19**, 587-595 (2016).

5. Murphy KR*, et al.* Postprandial sleep mechanics in Drosophila. *Elife* **5**, (2016).

6. Senapati B*, et al.* A neural mechanism for deprivation state-specific expression of relevant memories in Drosophila. *Nat Neurosci* **22**, 2029-2039 (2019).

7. Lee JH, Bassel-Duby R, Olson EN. Heart- and muscle-derived signaling system dependent on MED13 and Wingless controls obesity in Drosophila. *Proc Natl Acad Sci U S A* **111**, 9491-9496 (2014).

8. Zandawala M*, et al.* Modulation of Drosophila post-feeding physiology and behavior by the neuropeptide leucokinin. *PLoS Genet* **14**, e1007767 (2018).

9. Schiemann R, Lammers K, Janz M, Lohmann J, Paululat A, Meyer H. Identification and In Vivo Characterisation of Cardioactive Peptides in Drosophila melanogaster. *Int J Mol Sci* **20**, (2018).

10. Yu Y*, et al.* Regulation of starvation-induced hyperactivity by insulin and glucagon signaling in adult Drosophila. *Elife* **5**, (2016).

11. Guevara A, Gates H, Urbina B, French R. Developmental Ethanol Exposure Causes Reduced Feeding and Reveals a Critical Role for Neuropeptide F in Survival. *Front Physiol* **9**, 237 (2018).

12. Ameku T*, et al.* Midgut-derived neuropeptide F controls germline stem cell proliferation in a mating-dependent manner. *PLoS Biol* **16**, e2005004 (2018).

13. Tsao CH, Chen CC, Lin CH, Yang HY, Lin S. Drosophila mushroom bodies integrate hunger and satiety signals to control innate food-seeking behavior. *Elife* **7**, (2018).

14. Klose M, Shaw P. Sleep-drive reprograms clock neuronal identity through CREB-binding protein induced PDFR expression. *bioRxiv*, 2019.2012.2012.874628 (2019).

15. King AN*, et al.* A Peptidergic Circuit Links the Circadian Clock to Locomotor Activity. *Curr Biol* **27**, 1915-1927 e1915 (2017).

16. Cannell E, Dornan AJ, Halberg KA, Terhzaz S, Dow JAT, Davies SA. The corticotropin-releasing factor-like diuretic hormone 44 (DH44) and kinin neuropeptides modulate desiccation and starvation tolerance in Drosophila melanogaster. *Peptides* **80**, 96-107 (2016).
