# Supplementary material for: A neuronal ensemble encoding adaptive choice during sensory conflict in Drosophila
Source: Nat Commun. 2021 Jul 5;12:4131. doi: 10.1038/s41467-021-24423-y (PMC8257655; doi:10.1038/s41467-021-24423-y)
Supplement: Supplementary file 3 — Reporting summary [file 41467_2021_24423_MOESM3_ESM.pdf]

nature research

Corresponding author(s): Michael N. Nitabach

Last updated by author(s): Jun 3, 2021

Reporting Summary

Nature Research wishes to improve the reproducibility of the work that we publish. This form provides structure for consistency and transparency in reporting. For further information on Nature Research policies, see our Editorial Policies and the Editorial Policy Checklist.

Statistics

For all statistical analyses, confirm that the following items are present in the figure legend, table legend, main text, or Methods section.

n/a	Confirmed	
[ ]	[x]	The exact sample size (*n*) for each experimental group/condition, given as a discrete number and unit of measurement
[ ]	[x]	A statement on whether measurements were taken from distinct samples or whether the same sample was measured repeatedly
[ ]	[x]	The statistical test(s) used AND whether they are one- or two-sided<br>*Only common tests should be described solely by name; describe more complex techniques in the Methods section.*
[ ]	[x]	A description of all covariates tested
[ ]	[x]	A description of any assumptions or corrections, such as tests of normality and adjustment for multiple comparisons
[ ]	[x]	A full description of the statistical parameters including central tendency (e.g. means) or other basic estimates (e.g. regression coefficient) AND variation (e.g. standard deviation) or associated estimates of uncertainty (e.g. confidence intervals)
[ ]	[x]	For null hypothesis testing, the test statistic (e.g. *F*, *t*, *r*) with confidence intervals, effect sizes, degrees of freedom and *P* value noted<br>*Give P values as exact values whenever suitable.*
[x]	[ ]	For Bayesian analysis, information on the choice of priors and Markov chain Monte Carlo settings
[x]	[ ]	For hierarchical and complex designs, identification of the appropriate level for tests and full reporting of outcomes
[ ]	[x]	Estimates of effect sizes (e.g. Cohen's *d*, Pearson's *r*), indicating how they were calculated

Our web collection on statistics for biologists contains articles on many of the points above.

Software and code

Policy information about availability of computer code

Data collection	Zen v1.1.2.0 software was used to acquire all calcium imaging data. Pointgrey software was used to acquire videos of behaving flies. NanoDrop 2000 spectrophotometer was used to acquire food dye absorbance.
Data analysis	ImageJ/Fiji was used to define regions-of-interest (ROIs) and background subtraction for the analysis of all calcium imaging data. MATLAB code was used to analyze calcium imaging data. Caltech FlyTracker was used to track animals from videos. MATLAB code was used to analyze animal tracking data. neuprintExplorer was used to visualize EM data and neuprint-python code was used to access and analyze EM data. Some statistical tests were performed using built-in functions in Python and MATLAB. All custom written code is available from the corresponding author upon request. Standard curves for food intake analysis were calculated in GraphPad Prism v8.4.0 and v9.1.0. Graphpad Prism v8.4.0 and v9.1.0 was used to perform most statistical analyses.

For manuscripts utilizing custom algorithms or software that are central to the research but not yet described in published literature, software must be made available to editors and reviewers. We strongly encourage code deposition in a community repository (e.g. GitHub). See the Nature Research guidelines for submitting code & software for further information.

Data

Policy information about availability of data

All manuscripts must include a data availability statement. This statement should provide the following information, where applicable:

- Accession codes, unique identifiers, or web links for publicly available datasets
- A list of figures that have associated raw data
- A description of any restrictions on data availability

All data generated and used in this study are provided in the Source Data file.

Field-specific reporting

Please select the one below that is the best fit for your research. If you are not sure, read the appropriate sections before making your selection.

☒ Life sciences ☐ Behavioural & social sciences ☐ Ecological, evolutionary & environmental sciences

For a reference copy of the document with all sections, see nature.com/documents/nr-reporting-summary-flat.pdf

Life sciences study design

All studies must disclose on these points even when the disclosure is negative.

Sample size	No statistical methods were used to predetermine sample size. We chose sample sizes that are comparable to similar experiments conducted by others in the field, e.g., Lewis et al. Current Biology, 2015.
Data exclusions	No data were excluded.
Replication	Replication was extensive throughout the manuscript. Key experimental results were repeated independently at least twice. For each genotype/condition for all calcium imaging and behavioral experiment datasets, data was collected on at least two different days. Data collected on different days did not vary considerably. All attempts at replication were successful.
Randomization	In all experiments individual flies or groups of flies were randomly assigned to experimental groups.
Blinding	Investigators were not blinded during data collection since this was logistically not possible. This did not affect the outcome of any experiments since animals were randomly assigned prior to the start of all experiments.

Reporting for specific materials, systems and methods

We require information from authors about some types of materials, experimental systems and methods used in many studies. Here, indicate whether each material, system or method listed is relevant to your study. If you are not sure if a list item applies to your research, read the appropriate section before selecting a response.

Materials & experimental systems

n/a	Involved in the study	
☒	☐	Antibodies
☒	☐	Eukaryotic cell lines
☒	☐	Palaeontology and archaeology
☐	☒	Animals and other organisms
☒	☐	Human research participants
☒	☐	Clinical data
☒	☐	Dual use research of concern

Methods

n/a	Involved in the study	
☒	☐	ChIP-seq
☒	☐	Flow cytometry
☒	☐	MRI-based neuroimaging

Animals and other organisms

Policy information about studies involving animals; ARRIVE guidelines recommended for reporting animal research

Laboratory animals	For all experiments, 3-5 day old Drosophila melanogaster flies were used. For behavioral experiments, mixed-sex groups of flies were used. For calcium imaging experiments, female flies were used. For electron microscopy analyses, the data is from a female fly.
Wild animals	This study did not involve wild animals.
Field-collected samples	This study did not involve field-collected samples.
Ethics oversight	No ethical approval or guidance was required for the study protocol, as is common in experiments involving Drosophila flies.

Note that full information on the approval of the study protocol must also be provided in the manuscript.
